# Production of Margarine Fat Containing Medium- and Long-Chain Triacylglycerols by Enzymatic Interesterification of Peony Seed Oil, Palm Stearin and Coconut Oil Blends

**DOI:** 10.3390/foods13091405

**Published:** 2024-05-02

**Authors:** Man Zhang, Baocheng Xu, Dongkun Zhao, Mengqi Shen, Mengjie Li, Donghao Liu, Lili Liu

**Affiliations:** 1 College of Food and Bioengineering, Henan University of Science and Technology, Luoyang 471003, China; man56089@163.com (M.Z.); KUN18238796961@163.com (D.Z.); smq18339908215@163.com (M.S.); mj19981216@163.com (M.L.); 13592575978@163.com (D.L.); yangliuyilang@126.com (L.L.); 2 Henan International Joint Laboratory of Food Green Processing and Safety Control, Luoyang 471003, China; 3 National Experimental Teaching Demonstration Center of Food Processing and Safety, Henan University of Science and Technology, Luoyang 471003, China; 4 Henan Engineering Research Center of Food Material, Luoyang 471003, China

**Keywords:** peony seed oil, interesterification, Lipozyme TL IM, margarine, coconut oil, α-linolenic acid, MLCTs

## Abstract

This paper reports the preparation of margarine fat using Lipozyme TL IM as a catalyst and peony seed oil (PSO), palm stearin (PS) and coconut oil (CO) as raw materials. The results indicate that there were no significant changes in fatty acid composition before or after interesterification of the oil samples. However, the total amount of medium- and long-chain triglycerides (MLCTs) increased from 2.92% to 11.38% in sample E1 after interesterification, mainly including LaLaO, LaMO, LaPM, LaOO, LaPO and LaPP. Moreover, the slip melting point (SMP) of sample E1 decreased from 45.9 °C (B1) to 33.5 °C. The solid fat content (SFC) of all the samples at 20 °C was greater than 10%, indicating that they could effectively prevent oil exudation. After interesterification, the samples exhibited a β′ crystal form and could be used to prepare functional margarine.

## 1. Introduction

Traditionally, margarines with different plasticity and melting points have been prepared using partially hydrogenated vegetable oils. However, the hydrogenation of oil easily triggers the isomerization of cis-fatty acids, resulting in a generally high content of trans-fatty acids (TFAs) [1]. Many studies have demonstrated that TFAs are harmful to human health and can lead to obesity [2], cardiovascular disease [3], diabetes [4], depression [5] and other diseases. Furthermore, the addition of hydrogenated vegetable oil with considerable amounts of saturated fatty acid in the preparation of margarine also affects its taste and reduces its nutritional value, making it unable to meet the demand for nutritious and healthy food. To produce nutritious and healthy margarine, functional plastic fats (free of TFAs and with suitable contents of polyunsaturated fatty acids and structured lipids) should first be developed.

In recent years, researchers have performed several studies on the preparation of new margarine fats. Nutrition studies have shown that oils with balanced fatty acid (FA) content are beneficial to human health [6,7]. The related research on margarine has focused mainly on solving the problems of the single FA composition and low nutritional value of its base stock due to the excessive use of animal fat and hydrogenated plant oils. By combining soybean oil, palm stearin (PS) and coconut stearin through enzymatic interesterification (EI), Lakum and Ruttiya [8] produced margarine fats dominated by lauric, palmitic and oleic acids. Zhao and Li [9] used the lipase RM IM to catalyze the synthesis of medium- and long-chain triglycerides (MLCTs) rich in capric acid, lauric acid and oleic acid using camphora seed oil and camellia oil, which can be used for the preparation of nutritional margarine. Li and Jun [10] obtained margarine fat with a more diverse fatty acid composition using interesterification of butter and coconut oil. Related research on new margarine has increased the content of oleic acid, linoleic acid or lauric acid to a certain extent, but this strategy does not consider the current need to supplement α-linolenic acid. However, the diversity and distribution of fatty acids in triglycerides (TAGs) not only affect the crystallization characteristics but also affect the polymorphism of interesterified oils. For example, the carbon chain length of fatty acids in palm oil is between C12 and C20, which makes it crystallize more easily in β′ form [11]. The simultaneous introduction of medium-chain fatty acids (MCFAs) and α-linolenic acid into a new margarine fat by EI can not only change the FA distribution of TAGs but also greatly enhance the diversity of fatty acid types. However, whether this interesterified oil can meet the good physical and chemical requirements of margarine base stock warrants further study.

Peony seed oil (PSO), which originates and is produced in China, can be used as an edible and medicinal oil. The total unsaturated fatty acid content of PSO is higher than 85%, especially α-linolenic acid, which accounts for up to 40% [12]. Alpha-linolenic can effectively reduce plasma cholesterol and liver lipids and is beneficial for regulating the intestinal microbiota related to cholesterol metabolism [13]. Additionally, the total sterol content and tocopherols present in PSO are 372.65 mg/100 g and 53.73 mg/100 g, respectively [14]. It also contains natural pharmacologically active components such as stilbenes and flavonoids [15]. The unique fatty acid composition and diverse bioactive components make PSO possess medicinal and health benefits, such as enhanced immunity, anti-tumor properties and anti-oxidative properties [16]. In 2011, China’s Ministry of Health sanctioned PSO as a new source food, indicating a broad prospect for PSO to be used for food production in the future. Coconut oil (CO) is rich in MCFAs. MCFAs have a relatively small molecular weight and can be directly absorbed by the intestine and help the body absorb nutrients rather than storing them in the form of fat [17].

**Figure 1:**
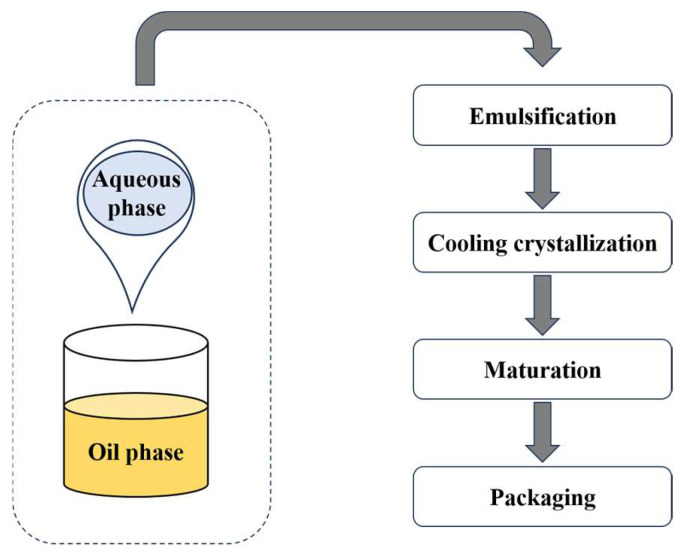
Margarine production process.

The aim of this work was to prepare trans fat-free margarine fats containing MLCTs and a certain amount of α-linolenic acid using a mixture of PSO, palm stearin (PS) and CO by interesterification using Lipozyme TL IM. The main processing steps of margarine include preparation of the water phase and oil phase, emulsification, cooling crystallization, maturation and packaging, as shown in Figure 1. To prepare margarine with suitable plasticity, pleasant mouthfeel and ideal crystal structure, we should first prepare a base stock with an appropriate melting point, low solid fat content (SFC) at body temperature and balanced fatty acid composition. In this study, PSO, PS and CO were used as raw materials for EI. The effects of EI on the physical and chemical properties (fatty acid and TAG composition, SFC, thermodynamic properties, etc.) of margarine base stock were investigated, and a new type of nutritional and healthy margarine fat was developed.

## 2. Materials and Methods

### 2.1. Materials

Peony seed oil (PSO) was obtained from Luoyang Guohuafang Peony Biotechnology Co., Ltd. (Luoyang, China), which was produced by mechanical pressing and refining technology. Palm stearin (PS) was obtained from Fengyi Oil Technology Co., Ltd. (Shanghai, China). Coconut oil (CO) was obtained from Yezhengdao Coconut Processing Professional Cooperative (Wenchang, China). Lipozyme TL IM (250 IUN/g) was obtained from Beijing Cliscent Technology Co., Ltd. (Beijing, China). Isopropanol (chromatographic grade), acetonitrile (chromatographic grade) and other analytical grade reagents were obtained from Tianjin Kemiou Chemical Reagent Co., Ltd. (Tianjin, China).

### 2.2. Fat Blending

PS has a high melting point due to its unique fatty acid composition. Adding a certain amount of PS can give margarine products ideal plasticity and good stability, so it is widely used in the preparation of margarine. In order to increase the nutritional value of margarine and improve its plasticity and stability, binary mixtures of PSO and PS were prepared according to the percentage of PSO in PS, that is, 40%, 35%, 30%, 25% and 20% (*w*/*w*) PSO in binary mixtures. Further, CO (10 wt% of total weight of PSO and PS) was mixed with the above binary mixtures, and the five ternary mixtures were named B1, B2, B3, B4 and B5, respectively.

### 2.3. Enzymatic Interesterification

First, 11.0 g of the ternary mixtures prepared by PSO, PS and CO was weighed and added to a reaction flask. Lipozyme TL IM (5 wt % of the ternary mixtures) was used as a biocatalyst and added to the ternary mixtures. The mixed matrix was reacted at a stirring rate of 1000 r.p.m. in a 60 °C water bath for 4 h. When the reaction was finished, the mixtures were centrifuged at 5000 r.p.m. for 1 min to separate the enzyme from the oil. The obtained samples were stored at 4 °C in darkness for subsequent analysis. The interesterified oils obtained by enzymatic interesterification reaction (EIE) of B1, B2, B3, B4 and B5 were named E1, E2, E3, E4 and E5, respectively.

### 2.4. Fatty Acid Composition

In a 10-mL plugged glass test tube, 0.1 g of oil sample was precisely weighed, followed by the addition of 0.8 mL of 2 mol/L potassium hydroxide methanol solution. The mixture was then subjected to ultrasonication under 50 °C for 10 min. Then, n-hexane (4 mL) was added, and the mixture was fully vortexed for approximately 60 s. After the solution was layered, the upper n-hexane phase was collected, and 0.5 g of anhydrous sodium sulfate was added to it. Then, the mixture was vortexed for 30 s, and the supernatant was collected for further analysis after centrifugation. In this study, the individual fatty acid content was determined according to the procedures described in GB 5009.168-2016 [18], and the TFAs were determined according to the procedures described in GB 5009.257-2016 [19].

### 2.5. Triacylglycerol Composition

The TAG composition of the samples E1, E2, E3, E4 and E5 were analyzed by a liquid chromatograph-mass spectrometer (LC-MS) (H-Class/TQ-SMicro, Waters Corporation, Milford, Massachusetts, USA) equipped with C18 (BEH) column of 2.1 × 50 mm i.d. (Waters Corporation, USA). Chromatographic grade isopropanol and acetonitrile were mixed at a volume ratio of 90:10 and used as mobile phase A. Ultrapure water and chromatographic grade acetonitrile were mixed at a volume ratio of 40:60 and used as mobile phase B. TAGs were separated using the following procedure: 0~15 min, 75~90% A; 15~20 min, 90% A; 20~32 min, 75% A. The injection volume was 1 μL, and the flow rate was 0.1 mL/min. The column temperature was set at 30 °C. All TAG contents are presented by percentage.

### 2.6. Peroxide Value

Peroxide value (PV) was determined by the acetic acid-isooctane method according to AOCS Cd 8b-90 [20].

### 2.7. Slip Melting Point

Slip melting point (SMP) was determined using open capillary tubes in accordance with the standard procedure [21].

### 2.8. Solid Fat Content

Solid fat content (SFC) of oils was determined using pulsed nuclear magnetic resonance (Minispec-mq20, Bruker, Billerica, Massachusetts, Germany) in accordance with AOCS Method Cd 16b-93 [22].

### 2.9. Melting and Crystallization Characteristics

The thermodynamic behavior of oils was determined by differential scanning calorimeter (DSC) (DSC1, Mettler-Toledo, Zurich, Switzerland). Using the empty crucible as a reference, oil samples of about 8~10 mg were weighed in an aluminum crucible for testing. Each sample was heated from 25 °C to 80 °C at a rate of 40 °C/min, followed by a cooling process from 80 °C to −50 °C and then heated from −50 °C to 80 °C at a rate of 5 °C/min. The temperature was maintained for 10 min between each step to obtain thermograms of crystallization and melting.

### 2.10. Polymorphism

The polymorphic structure of the oil samples was determined by X-ray diffraction (XRD) (SmartLab SE, Rigaku Corporation, Tokyo, Japan). The melted oil samples were poured into plastic molds and kept at 25 °C for 12 h before measurement (short spacing: 5° < 2θ < 40°; scanning rate: 2°/min; step width 0.02°).

### 2.11. Statistical Analysis

Each experiment was conducted in triplicate, and the results were presented as means ± standard deviations. Experimental data were processed by Microsoft Excel 2019, SPSS 26.0, and Origin 2018. Variance (ANOVA) was used for statistical analysis of differences. Significance was compared using Duncan’s test, with a threshold of *p* < 0.05.

## 3. Results and Discussion

### 3.1. Fatty Acid Composition

The fatty acid compositions of PSO, PS, CO and their mixtures in different proportions are shown in Table 1. After interesterification, the fatty acid distribution in TAGs changed (Table 2), but the fatty acid composition of the whole oil sample remained unchanged. This result can be attributed to the fact that during interesterification, fatty acids detached from the glycerol backbone with the catalysis of lipases. Subsequently, the vacated position on the glycerol backbone of triglycerides was randomly occupied by another free fatty acid. This process resulted in a redistribution of fatty acids within triglycerides, while the FA composition of the oil sample remained unchanged [23]. Similar results were also obtained in the study by Lakum and Ruttiya [8], in which soybean oil and coconut stearin were used to prepare palm-based margarine fat by interesterification. As shown in Table 1, CO was dominated by saturated fatty acids, with a saturation of up to 90.76%. Lauric acid is the main MCFA in CO, accounting for 35.89% of total fatty acids. Lauric acid is one of the main active components of CO, which exerts antibacterial and antiviral effects and prevents cardiovascular diseases [24]. In PSO, the contents of polyunsaturated α-linolenic acid and linoleic acid were 36.80% and 26.80%, respectively. As an essential fatty acid, α-linolenic acid must be obtained via the diet. It can reduce blood lipids and blood pressure, protect nerve tissue, prevent allergic diseases and inhibit tumor cell metastasis [25]. In addition, PS is rich in saturated fatty acids, exhibits a high melting point and is often used in margarine to adjust the plasticity of the product. In all samples, no TFAs were detected. In our study, three types of oil were mixed (see the Fat Blending section) at a specific ratio to obtain margarine fats with low saturation (unsaturated fatty acid content of 47.66~56.36%), reasonable fatty acid composition and high nutritional value. Singh and Chopra [26] also synthesized structured lipids using perilla seed oil and palm olein as raw materials and Lipozyme TL IM as catalyst. The structured lipids produced using a 70:30 (palm olein: perilla seed oil) substrate molar ratio had suitable omega-6/omega-3 fatty acid ratio and balanced fatty acid composition. These omega-3 FA-rich products with desirable physicochemical properties can meet people’s demands regarding a healthy life.

### 3.2. Triacylglycerol Composition

TAGs are the main components of oil, and each oil has a unique TAG composition. PSO, PS and CO, along with representative physical mixture oil sample (B1) and interesterified sample (E1), have different TAG compositions, as shown in Table 2. In accordance with the saturation degree of fatty acids in TAGs, these can be categorized into four types: S_3_, S_2_U, SU_2_ and U_3_ (S denotes saturated fatty acids while U denotes unsaturated fatty acids). U_3_ TAG mainly existed in PSO, including LnLO (8.98%), LnLO (8.86%) and LnOO (7.14%), which can be used to improve the softness of the sample. The main TAGs in PS were PPO (26.21%), PPL (9.38%) and PPP (8.68%). S_3_ and S_2_U TAGs contribute to the plastic structure of the sample. Due to the redistribution of fatty acids within and between TAGs, the composition of TAGs changes significantly. Compared with those in sample B1, the content of short-chain triglycerides in E1 decreased; for example, CaCaLa and CaCaLa decreased from 1.82% and 1.97% to 0.04% and 0.08%, respectively. The contents of long-chain TAGs such as LnLn, LLL and PPP also decreased from 2.17%, 2.25% and 11.03% to 0.12%, 0.80% and 6.72%, respectively. Additionally, the contents of the S_3_ and U_3_ TAGs decreased from 23.07% and 26.90% to 14.59% and 16.05%, respectively, while the contents of the S_2_U and SU_2_ TAGs increased from 25.21% and 24.82% to 30.81% and 37.55%, respectively. This result was consistent with the change in TAG types observed during the interesterification of PS and soybean oil [27]. Specifically, the total content of the MLCTs increased from 2.92% to 11.38 8%, including LaMO (from 0.17% to 0.47%), LaOO (from 0.05% to 1.40%), LaPO (from 0.14% to 3.93%) and LaPP (from 0.17% to 3.59%). MLCTs have functional properties, such as preventing obesity and alleviating plasma lipid disorders [28,29].

### 3.3. Peroxide Value

**Figure 2:**
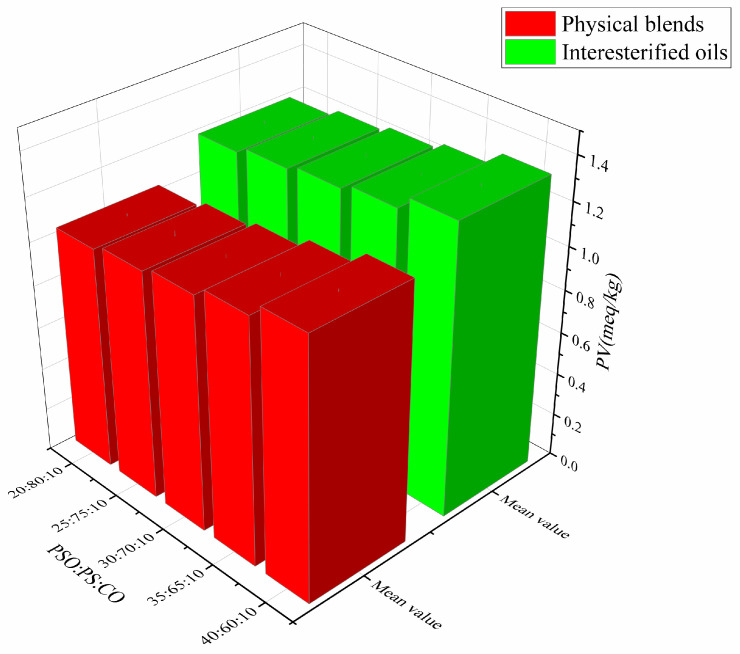
Peroxide value of physical blends and interesterified oil samples.

Lipid oxidation is a main cause of food spoilage [30], which not only generates off-flavors and destroys nutrients and bioactive compounds, but also forms potentially toxic compounds [31]. PV is used as an important indicator to evaluate the oxidation of fats and oils. The PVs of the mixed oils before and after interesterification are shown in Figure 2. The PVs of physical ternary mixtures were slightly lower than those of the corresponding interesterified oils, which is similar to the findings reported by Adhikari and Hu [32]. The reason for this may be attributed to the fact that the mixed oil itself contained a certain amount of natural antioxidants, such as tocopherols, which would protect oil from oxidation [33]. Generally, edible oils with a PV below 10 meq/kg are considered safe for consumption. In this study, the PVs of all the oil samples before and after interesterification were below 4 meq/kg, indicating that the interesterified oils are still fresh and healthy. Related studies have also demonstrated that interesterification has minimal impact on oil oxidative stability [26]. Therefore, EIE could be used as a reliable technology to produce trans-free structured lipids rich in α-linolenic acid.

### 3.4. Slip Melting Point and Solid Fat Content

**Figure 3:**
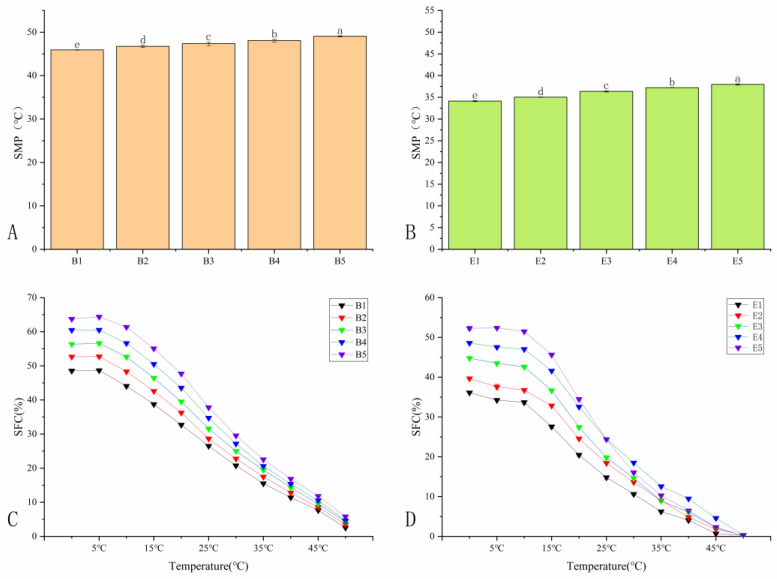
Slip melting point of the oil samples (**A**) before and (**B**) after interesterification; solid fat content (%) of the oil samples (**C**) before and (**D**) after interesterification. Different lowercase letters indicate significant differences (*n* = 3, *p* < 0.05).

The changes in SMP of the samples before and after interesterification are shown in Figure 3A,B. As indicated in Table 2, PS is rich in long-chain TAGs like PPO (26.21%) and PPP (8.68%), contributing to its high melting point. In the mixed oil samples, the SMP of B1–B5 exhibited a positive correlation with the PS content. Although the SMP of E1–E5 decreases after interesterification, it still follows this trend. In particular, the SMP of the physical blend oil decreased from 45.93 °C for sample B1 to 33.53 °C for interesterified sample E1, indicating that it had benign adjustability after EI. A similar result was also reported by Adhikari and Shin [34], in which they studied rice bran oil, and PS and CO were used to prepare a margarine base stock.

The changes in the SFC of the samples are depicted in Figure 3C,D. In addition to SMP, various functional properties of oils, including sensory properties and extensibility, may be related to SFC [35,36]. Figure 3C,D show that there were obvious differences in the SFC curves of B1–B5 and E1–E5. During the heating process from 5 °C to 35 °C, the SFC of both the physically mixed oils and the interesterified oils decreased rapidly. The SFC of sample B1 decreased from 48.92% to 15.47%, and the SFC of sample E1 decreased from 34.35% to 6.48%. At a low temperature of 10 °C, the SFC of samples E1 was 33.61%, indicating that the interesterified sample had good ductility. At 20 °C, the SFC of sample E1 was 20.01%, which was greater than 10%, indicating that it could prevent oil exudation well and had good stability [37]. SFC in the temperature range of 35–37 °C affected the thickness and flavor release [38]. At 35 °C, the SFC of sample E1 was 6.47%, which was significantly lower than that of sample B1 (15.47%), indicating that the interesterified sample could quickly melt in the mouth and provide a good taste. The results showed that E1 had good anti-oil permeability and improved taste and has potential application prospects in the development of food-specific oils.

### 3.5. Melting and Crystallisation Characteristics

**Figure 4:**
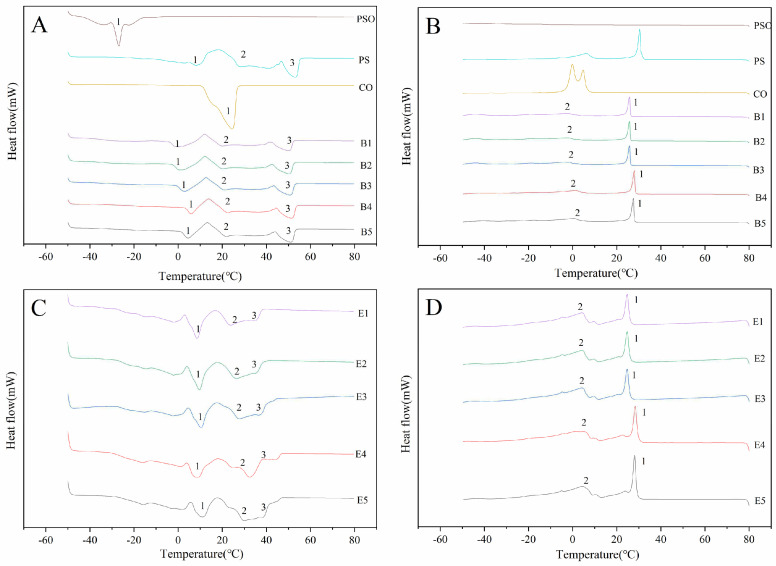
Melting thermograms of (**A**) PSO, PS, CO, physical blends and (**C**) interesterified oil samples; crystallization thermograms of (**B**) PSO, PS, CO, physical blends and (**D**) interesterified oil samples. The peaks 1, 2 and 3 in Figure 4A,C represent the endothermic peaks in the melting process of the samples from −60 °C to 80 °C. The peaks 1 and 2 in Figure 4B,D represent the exothermic peaks in the crystallization process of the samples from 80 °C to −60 °C.

The melting curves of PSO, PS, CO and their mixtures before and after interesterification are shown in Figure 4A,C. Melting characteristics are an important indicator to evaluate the flavor release of plastic fats such as margarine [39]. As shown in Figure 4A, PSO and CO have strong endothermic peaks at −26.8 °C and 24.4 °C, respectively. It shows that the melting point of PSO is very low and it is liquid at room temperature, which is related to the large amount of linoleic acid (26.85%) and α-linolenic acid (36.27%) in PSO (Table 1). CO melted at approximately 20 °C, corresponding to its endothermic peak. There are three endothermic peaks for PS at 7.9 °C, 26.9 °C and 53.2 °C (Figure 4A). The sharp endothermic peak at 53.2 °C indicated that PS contains a large amount of long-chain TAGs, such as PPO (26.21%), POS (10.00%) and PPP (8.68%), which have a high melting point. The melting curves of samples B1–B5 have three obvious endothermic peaks and the interesterified samples E1–E5 also have three obvious endothermic peaks. The endothermic peak of B1 at 52 °C was reduced to 37 °C for E1 after interesterification. These changes in thermodynamic properties were caused by the change of TAG types in interesterification [40]. The content of high melting point long chain TAG decreased, while the content of medium melting point S_2_U and SU_2_ TAG increased.

The crystallization curves of PSO, PS, CO and their mixtures before and after interesterification are shown in Figure 4B,D. There were two main exothermic peaks in the five different proportions of the oil mixtures: sharp peak 1 and relatively flat peak 2. For samples E1–E5, with increasing PS ratios, the saturated TAG content increased gradually, and the crystallization temperature also increased. Similarly, the changes in the crystallization temperature of interesterified oil samples E1–E5 were the same. However, compared with that before interesterification, the crystallization starting point of samples E1–E5 was slightly lower than that of the physical blends because the TAG composition of the oil has an important influence on its crystallization. During the crystallization process, S_3_ TAG crystallized first, followed by S_2_U, SU_2_ and U_3_ TAGs [41]. After interesterification, the content of S_2_U and SU_2_ TAG increased from 25.21% and 24.82% to 30.81% and 37.55%, respectively, resulting in a delay in the crystallization starting point. Moreover, the area of the high melting point peak 1 of E1–E5 was smaller than that of B1–B5, and the peak width of the crystallization peaks increased slightly, which means that the plasticity of the oil increased and was more conducive to the production of special oils such as margarine.

### 3.6. Polymorphism

**Figure 5:**
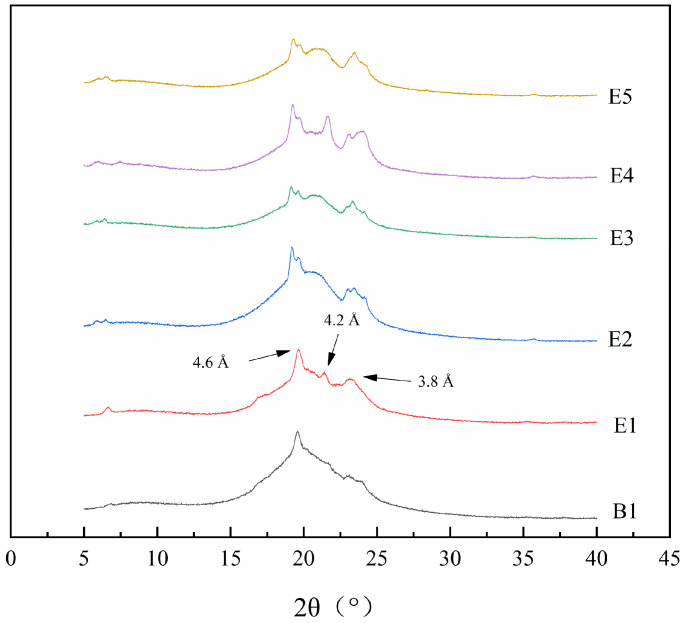
X-ray diffraction patterns of the oil samples before and after interesterification.

The performance of oil is closely related to its polymorphic structure. In an oil system, there are three types of polymorphic forms, that is, α, β′ and β, which are generated according to the different growth environments of the crystals [42]. X-ray diffraction is frequently employed to determine the polymorphic structure of lipids. The polymorphic forms of interesterified oils with different ratios of PSO, PS and CO were studied, and the results are shown in Figure 5. There were two types of diffraction peaks in sample B1: one was the β form at a short spacing of 4.6 Å, and the other was the β′ form at 3.8 Å. The crystal form of the interesterified sample (E1) was similar. In addition to 3.8 Å and 4.6 Å, the β′ form also appeared at 4.2 Å. This indicates that the interesterification promoted the transformation of the β form to the β′ form in oil. For plastic fats such as margarine, due to the small size of the β′ crystal and the large specific surface area, a delicate crystal network forms easily, which provides a better oral sensation [43]. The β′ type crystals of interesterified samples obtained in our study increased, indicating that this material is suitable for the production of margarine. Similarly, a plastic fat prepared using beef tallow, PS, and camellia oil also exhibited optimal β′ crystal [44]. Additionally, Xue and Wang [45] employed chemical interesterification to prepare a nutritional plastic fat rich in linolenic acid using lard and perilla seed oil. The results showed that when the ratio of lard to perilla seed oil was set as 8:2 and 7:3, the content of β′ crystal presented in the interesterified fats was as high as 67.76% and 74.30%, and the crystal was small and uniform, indicating they were suitable for the production of margarine.

## 4. Conclusions

In this study, plastic fat containing lauric acid and α-linolenic acid was produced using Lipozyme TL IM as a catalyst for the interesterification of PSO, PS and CO. In the obtained mixed oils, the content of unsaturated fatty acids was greatly improved and was 56.36% of the total fatty acids in sample B1. Moreover, the MLCT content increased from 2.92% in sample B1 to 11.38% in sample E1, which could provide nutritional benefits for human health. In addition, the SMP of sample E1 decreased from 45.93 °C (B1) to 33.53 °C after interesterification, and the SFC at 20 °C was greater than 10%, indicating that its plasticity improved and that it was stable enough to prevent oil exudation. Sample E1, with a ratio of PSO, PS and CO of 40:60:10, crystallized in the β′ form, which is an ideal crystal form for the production of margarine. Overall, sample E1 has the most potential to be used as a new type of margarine fat containing high contents of MLCTs and α-linolenic acid. Furthermore, using other linolenic acid-rich edible oils that share similar fatty acid and triglyceride profiles with PSO as raw materials, it is possible to obtain modified oils with comparable physicochemical properties through interesterification. This will contribute to the development of new types of nutritional and healthy margarine.

## Figures and Tables

**Table 1:** Fatty acid composition of PSO, PS, CO and their physical blends.

FA (%)	PSO	PS	CO	B1	B2	B3	B4	B5
C6:0	ND	ND	0.64 ± 0.02 ^a^	ND	ND	ND	ND	ND
C8:0	ND	ND	8.88 ± 0.40 ^a^	0.37 ± 0.01 ^b^	0.41 ± 0.01 ^b^	0.37 ± 0.02 ^b^	0.37 ± 0.01 ^b^	0.43 ± 0.00 ^b^
C10:0	ND	ND	8.20 ± 0.42 ^a^	0.40 ± 0.02 ^b^	0.43 ± 0.02 ^b^	0.44 ± 0.06 ^b^	0.42 ± 0.01 ^b^	0.44 ± 0.00 ^b^
C12:0	ND	0.17 ±0.01 ^c^	35.89 ± 0.11 ^a^	3.69 ± 0.12 ^b^	3.80 ± 0.13 ^b^	3.79 ± 0.14 ^b^	3.90 ± 0.10 ^b^	3.90 ± 0.03 ^b^
C14:0	0.05 ± 0.00 ^d^	1.40 ± 0.03 ^c^	19.88 ± 1.11 ^a^	2.26 ± 0.16 ^b^	2.37 ± 0.08 ^b^	2.70 ± 0.04 ^b^	2.82 ± 0.01 ^b^	2.65 ± 0.04 ^b^
C16:0	6.69 ± 0.19 ^g^	48.34 ± 0.12 ^a^	11.87 ± 0.16 ^f^	29.94 ± 0.97 ^e^	32.40 ± 0.60 ^d^	32.43 ± 1.27 ^d^	34.15 ± 0.20 ^c^	36.66 ± 0.40 ^b^
C17:0	ND	0.18 ± 0.00 ^a^	ND	0.12 ± 0.01 ^cd^	0.12 ± 0.02 ^d^	0.14 ± 0.01 ^bc^	0.15 ± 0.00 ^b^	0.14 ± 0.01 ^bcd^
C18:0	4.58 ± 0.02 ^e^	8.63 ± 0.13 ^a^	5.27 ± 0.01 ^d^	6.45 ± 0.32 ^c^	6.30 ± 0.05 ^c^	7.49 ± 0.59 ^b^	7.63 ± 0.13 ^b^	7.62 ± 0.06 ^b^
C18:1	25.05 ± 0.10 ^e^	33.09 ± 0.21 ^a^	8.01 ± 0.10 ^f^	27.24 ± 0.44 ^d^	27.23 ± 0.21 ^d^	27.82 ± 0.44 ^cd^	28.17 ± 0.13 ^bc^	28.84 ± 0.64 ^b^
C18:2	26.80 ± 0.58 ^a^	7.57 ± 0.14 ^g^	1.23 ± 0.09 ^h^	14.35 ± 0.11 ^b^	13.58 ± 0.45 ^c^	12.73 ± 0.20 ^d^	11.90 ± 0.04 ^e^	10.96 ± 0.28 ^f^
C18:3	36.30 ± 0.31 ^a^	ND	ND	14.76 ± 0.30 ^b^	12.95 ± 0.27 ^c^	11.56 ± 0.38 ^d^	9.93 ± 0.05 ^e^	7.87 ± 0.06 ^f^
C20:0	0.24 ± 0.00 ^d^	0.62 ± 0.02 ^a^	0.13 ± 0.01 ^e^	0.41 ± 0.02 ^c^	0.40 ± 0.02 ^c^	0.53 ± 0.09 ^b^	0.57 ± 0.00 ^ab^	0.52 ± 0.00 ^b^
C22:0	0.30 ± 0.00 ^a^	ND	ND	ND	ND	ND	ND	ND
ΣUSFA	88.14 ± 0.17 ^a^	40.67 ± 0.08 ^g^	9.24 ± 0.03 ^h^	56.36 ± 0.64 ^b^	53.76 ± 0.51 ^c^	52.11 ± 0.64 ^d^	49.99 ± 0.04 ^e^	47.66 ± 0.37 ^f^

All values are mean ± SD of three analyses. ND, not detected; FA, fatty acid; PSO, peony seed oil; PS, palm stearin; CO, coconut oil. Different lowercase letters in the same line indicate significant differences (*n* = 3, *p* < 0.05).

**Table 2:** Triglyceride composition of PSO, PS, CO, sample B1 and E1.

TAG (%)	PSO	PS	CO	B1	E1
CaCaCa	ND	TD	2.58 ± 0.12	0.17 ± 0.04	TD
CaCaLa	ND	0.03 ± 0.00	17.89 ± 0.59	1.82 ± 0.11	0.04 ± 0.02
CaLaLa	ND	0.06 ± 0.02	18.85 ± 0.71	1.97 ± 0.13	0.08 ± 0.03
LnLnLn	5.61 ± 0.18	TD	TD	2.17 ± 0.06	0.12 ± 0.01
LnLnL	4.64 ± 0.15	TD	TD	1.67 ± 0.14	0.33 ± 0.03
LaLaM	ND	0.10 ± 0.02	22.94 ± 1.09	2.05 ± 0.15	0.12 ± 0.04
LnLL	5.94 ± 0.19	0.04 ± 0.01	TD	2.34 ± 0.10	0.94 ± 0.06
LnLnO	5.90 ± 0.21	0.02 ± 0.00	TD	2.40 ± 0.09	0.92 ± 0.06
LnLnP	2.71 ± 0.10	TD	TD	1.07 ± 0.04	1.04 ± 0.04
LaLaO	ND	0.02 ± 0.01	3.96 ± 0.18	0.26 ± 0.06	0.64 ± 0.04
LaMM	ND	0.06 ± 0.03	13.33 ± 0.44	0.93 ± 0.08	0.37 ± 0.02
LLL	5.80 ± 0.13	0.11 ± 0.02	TD	2.25 ± 0.15	0.80 ± 0.05
LnLnS	1.93 ± 0.09	TD	TD	0.62 ± 0.05	0.70 ± 0.02
LnLO	8.98 ± 0.21	0.14 ± 0.02	0.02 ± 0.00	3.56 ± 0.13	2.05 ± 0.17
LnLP	2.30 ± 0.08	0.06 ± 0.03	TD	0.90 ± 0.06	2.55 ± 0.13
PoLL	0.02 ± 0.00	0.02 ± 0.02	ND	0.02 ± 0.01	0.08 ± 0.04
LaMO	ND	ND	2.67 ± 0.20	0.17 ± 0.05	0.47 ± 0.03
LaPM	ND	ND	8.73 ± 0.31	0.58 ± 0.04	0.86 ± 0.14
LnLS	6.43 ± 0.17	0.28 ± 0.02	0.02 ± 0.01	1.18 ± 0.10	1.76 ± 0.11
LLO	8.86 ± 0.22	0.42 ± 0.02	0.03 ± 0.02	3.99 ± 0.29	2.52 ± 0.20
LnOO	7.14 ± 0.15	0.20 ± 0.01	0.02 ± 0.00	3.12 ± 0.20	2.67 ± 0.13
LnOP	3.64 ± 0.15	0.66 ± 0.05	0.02 ± 0.01	1.80 ± 0.12	6.54 ± 0.26
LLP	3.45 ± 0.07	1.30 ± 0.11	0.03 ± 0.02	2.01 ± 0.13	3.55 ± 0.11
LaOO	TD	ND	0.65 ± 0.05	0.05 ± 0.02	1.40 ± 0.07
LaPO	ND	ND	1.72 ± 0.13	0.14 ± 0.04	3.93 ± 0.25
MMO	ND	0.04	1.02 ± 0.09	0.08 ± 0.03	1.50 ± 0.09
LaPP	ND	ND	1.81 ± 0.13	0.17 ± 0.02	3.59 ± 0.23
LOO	3.57 ± 0.14	0.98 ± 0.08	0.03 ± 0.02	1.60 ± 0.09	2.09 ± 0.16
LLS	2.94 ± 0.09	0.22 ± 0.02	0.02 ± 0.00	0.84 ± 0.05	1.33 ± 0.06
LOP	3.15 ± 0.08	7.60 ± 0.54	0.16 ± 0.03	4.06 ± 0.19	6.95 ± 0.15
PoOO	1.04 ± 0.05	2.51 ± 0.15	0.05 ± 0.02	1.33 ± 0.07	2.33 ± 0.08
PPL	0.53 ± 0.04	9.38 ± 0.41	0.13 ± 0.01	4.42 ± 0.26	6.07 ± 0.29
POPo	0.40 ± 0.04	7.02 ± 0.35	0.35 ± 0.03	3.30 ± 0.26	4.30 ± 0.14
MPO	TD	2.29 ± 0.14	0.95 ± 0.08	0.97 ± 0.07	1.91 ± 0.15
OOO	4.85 ± 0.12	2.36 ± 0.09	0.06 ± 0.03	2.45 ± 0.11	2.19 ± 0.13
SOL	5.89 ± 0.14	2.85 ± 0.22	0.08 ± 0.04	2.82 ± 0.11	2.82 ± 0.20
POO	1.65 ± 0.07	9.25 ± 0.68	0.24 ± 0.07	4.59 ± 0.20	3.60 ± 0.17
PPO	0.68 ± 0.06	26.21 ± 1.07	1.05 ± 0.08	14.07 ± 0.69	12.80 ± 0.49
PPP	ND	8.68 ± 0.32	0.31 ± 0.07	11.03 ± 0.29	6.72 ± 0.27
SOO	1.34 ± 0.10	2.71 ± 0.11	0.06 ± 0.02	1.60 ± 0.05	1.03 ± 0.08
POS	0.37 ± 0.04	10.00 ± 0.53	0.15 ± 0.03	4.70 ± 0.18	3.28 ± 0.11
SPP	ND	3.15 ± 0.11	ND	3.86 ± 0.14	2.46 ± 0.15
SSO	0.17 ± 0.03	0.77 ± 0.04	0.02 ± 0.00	0.40 ± 0.03	0.22 ± 0.03
SSP	0.03 ± 0.01	0.43 ± 0.03	ND	0.46 ± 0.03	0.31 ± 0.02
SSS	TD	0.02 ± 0.01	TD	0.02 ± 0.01	0.02 ± 0.01
S_3_	0.04 ± 0.01	12.53 ± 0.39	86.44 ± 2.87	23.07 ± 1.07	14.59 ± 0.52
S_2_U	1.76 ± 0.11	48.70 ± 1.56	11.66 ± 0.38	25.21 ± 0.92	30.81 ± 2.01
SU_2_	35.83 ± 1.33	31.96 ± 0.89	1.64 ± 0.05	24.82 ± 1.12	37.55 ± 1.83
U_3_	62.37 ± 1.42	6.80 ± 0.37	0.26 ± 0.05	26.90 ± 0.78	17.05 ± 0.40
MLCT	ND	0.18 ± 0.04	55.80 ± 1.81	2.92 ± 0.13	11.38 ± 0.36

All values are mean ± SD of three analyses. TAG, triglyceride; PSO, peony seed oil; PS, palm stearin; CO, coconut oil Ca, capric acid; La, lauric acid; M, myristic acid; P, palmitic acid; Po, palmitoleic acid; S, stearic acid; O, oleic acid; L, linoleic acid; Ln, linolenic acid; TD, trace detected; ND, not detected.

## Data Availability

The original contributions presented in the study are included in the article, further inquiries can be directed to the corresponding author.
